# Agency judgments in post-stroke patients with sensorimotor deficits

**DOI:** 10.1371/journal.pone.0230603

**Published:** 2020-03-18

**Authors:** Yu Miyawaki, Takeshi Otani, Shu Morioka

**Affiliations:** 1 Graduate School of Health Science, Kio University, Kitakaturagi-gun, Nara, Japan; 2 Department of Rehabilitation Medicine, Keio University School of Medicine, Tokyo, Japan; 3 Department of Rehabilitation, Ishikawa Hospital, Himeji, Hyogo, Japan; 4 Neurorehabilitation Research Center, Kio University, Kitakaturagi-gun, Nara, Japan; University of Rome, ITALY

## Abstract

Sense of agency refers to the feeling of being in control of one’s actions. Previous research has demonstrated that sense of agency is produced through the sensorimotor system, which is involved in comparing internal predictions with sensory feedback in motor control. Therefore, sensorimotor deficits might impair agency through a sensorimotor system malfunction. The present study examined this hypothesis by investigating post-stroke patients who had suffered a subcortical stroke that damaged regions associated with sensorimotor function. To examine agency judgments with respect to motor control, we adopted a self-other attribution task and applied it to post-stroke patients. Participants traced a horizontal straight line and received visual feedback through a cursor on a monitor. The cursor movement reflected either the participants’ actual movement or the movement of an “other” that had been previously recorded. Participants judged whether the cursor movement reflected their own movement (self) or an other’s movement while they engaged in four cycles of the horizontal tracing movement. After each trial, participants reported their self-other judgment on a nine-point scale. Post-stroke patients completed the experiment with their paretic as well as their non-paralyzed upper limbs. Compared to healthy controls, patients made significantly more self-attributions of others’ movements. Interestingly, such misattributions were observed in the patients’ performance using both paretic and non-paralyzed upper limbs. These results suggest that post-stroke patients with sensorimotor deficits form misattributions that cannot be explained solely by the sensorimotor system’s role in motor control. We discuss these misattributions in post-stroke patients in terms of cue integration theory.

## Introduction

Sense of agency refers to the subjective experience of controlling one’s actions [1–4]. Typically, healthy individuals have no difficulty producing movement associated with voluntary action, but individuals with motor disorders often experience difficulty controlling their intended actions because their sense of agency may be altered. Previous studies have suggested that sense of agency is altered in patients with motor disorders, such as Parkinson’s disease [5], Gilles de la Tourette syndrome [6], or functional movement disorders [7,8], and that sense of agency is not always stable, but rather might fluctuate as a function of motor control.

In motor control, the prediction of action sensory feedback is based on efference copies of the motor commands produced in the brain [9–11]. This internal prediction works not only in motor control and motor learning, but also in the registration of agency [12,13], where it is compared with actual sensory feedback. When the prediction matches the sensory feedback, a sense of agency is produced through sensorimotor comparison [14]. Sense of agency may depend on the consistency between predicted and actual movement, i.e., prediction error [15]. Asai [16] confirmed this in a study where participants received visual feedback (cursor movement on a monitor) while tracing a target line. When the cursor feedback represented a fake movement that was spatiotemporally similar to the actual movement (i.e., a prerecorded self-movement), participants mistakenly attributed the fake movement to their own action. Conversely, when participants received cursor feedback representing a fake movement that was unlike their actual movement (i.e., a prerecorded other’s movement), no significant misattributions were observed. These results suggest that error detection in motor control plays an important role in self-other sensory attribution [17,18]. Therefore, the experience of agency might be altered by a malfunctioning sensorimotor system, which computes prediction error by comparing internal prediction with sensory feedback.

If the sensorimotor system contributes to the registration of agency, its corruption may result in disturbances in agency. However, the relationship between a malfunction in the sensorimotor system (i.e., defects in the error detection processes) and sense of agency remains unclear. Although previous research indirectly examined this relationship in healthy people by experimentally manipulating visual feedback [19], it is necessary to examine sense of agency in patients with motor disorders associated with the sensorimotor system. Several studies have suggested that patients with motor disorders, such as Parkinson’s disease [5], Gilles de la Tourette syndrome [6], or functional movement disorders [7,8] can experience an altered sense of agency. Such patients are known to have impairments in sensorimotor integration associated with sensory input and motor output central processing [20,21], which are reported to be related to their altered sense of agency [5,6,8,22]. If sensorimotor deficits cause such agency disturbances, the altered sense of agency might also be observed in patients with other motor disorders. Investigating this possibility could reveal the relationship between a sensorimotor system malfunction and an altered sense of agency; therefore, the present study focused on sensorimotor deficits in post-stroke patients.

Stroke is the most common disease to cause sensorimotor deficits, including hemiparesis, ataxia, and spasticity [23]. Moreover, strokes can often result in severe neurological dysfunction, such as apraxia or anosognosia, by causing extensive damage to cortical regions such as the parietal or premotor cortices [24–26]. Several studies have demonstrated that post-stroke patients with such neurological dysfunction have impaired motor awareness [27–29]. However, few studies have investigated whether post-stroke patients without such neurological dysfunction demonstrate an altered sense of agency. To examine the impact of sensorimotor deficits themselves, brain lesion sites should be considered. Recent meta-analysis studies have found that sense of agency is associated with premotor areas, the posterior insula, occipital lobe, and cerebellum, indicating that self-agency is associated with regions involved in motor control and sensory processing [30,31]. Although lesions in these areas might result in agency disturbances, they can also be associated with neurological dysfunctions, including anosognosia, which may obscure the relationship between sense of agency and sensorimotor deficits. To examine the impact of sensorimotor deficits, the present study recruited patients with a subcortical stroke, separate from these cortical areas.

Post-stroke patients often have difficulty exerting voluntary control over one side of their body and they receive insufficient sensory feedback regarding movement in their paretic limb because of sensorimotor deficits, which may also result in an altered sense of agency. Several studies have reported motor recovery related to sensorimotor deficits [32,33], but few have examined the impact of post-stroke sensorimotor deficits on sense of agency. If sensorimotor deficits affect the ability to compare internal predictions with sensory feedback, post-stroke patients would be expected to have difficulty identifying self versus other sensory attributions in their paretic limb movements. The present study aimed to examine this hypothesis by using Asai’s self-other attribution task [16] to investigate differences in paretic and non-paralyzed upper limbs in self-other attribution. We assumed that if post-stroke patients with sensorimotor deficits had error detection impairments, patients would formulate self-other attributions that differed from those of healthy elderly individuals while performing the task with their paretic upper limbs.

## Methods and materials

### Participants

**Fig 1:**
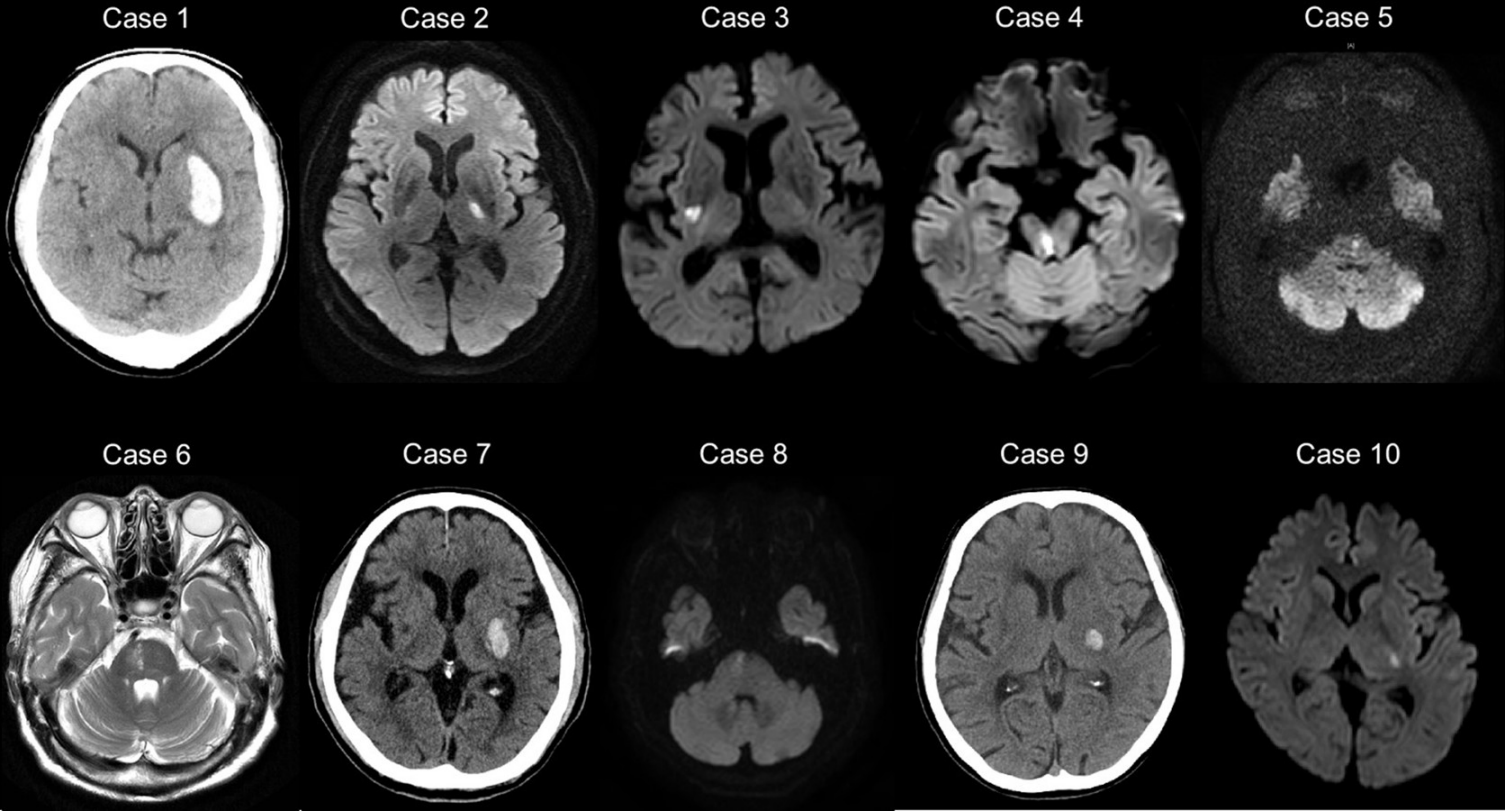
MRI/CT images presenting the lesion sites of 10 post-stroke patients.

A total of 10 post-stroke patients with sensorimotor deficits (mean age = 69.3 years, SD = 9.0, range = 54–86 years; four females) and 10 healthy elderly individuals with no history of any psychiatric or neurological disorders (mean age = 66.6 years, SD = 4.7, range = 57–75 years; six females) participated in this study. All participants were right-handed. All patients were hospitalized at the Jinjukai Ishikawa Hospital (Hyogo, Japan). Only patients who had been diagnosed with a subcortical stroke (i.e., no lesions in the cortical areas including the parietal region, prefrontal cortex, and insula) were included to exclude the presence of neurological dysfunction such as apraxia or anosognosia (Fig 1). Participants did not have any cognitive impairment diagnoses, such as dementia or other psychiatric or neurological disorders and were assessed for anosognosia through clinical interview to verify that they were aware of the sensorimotor deficit and to determine whether they could explain these deficits. They were also assessed with the Catherine Bergego Scale for neglect (Table 1).

**Table 1:** Summary of patients’ clinical characteristics.

Case	Lesion site	Time since lesion (days)	Paralysis side	BRS (upper limb)	Tactile sensation (upper limb)	Proprioceptive sensation (upper limb)		CBS (objective)	MMSE
						Shoulder	MCP		
1	Lt. putamen	112	Rt	V	2/5	Mild	Mild	1	30
2	Lt. IC	53	Rt	VI	4/5	Intact	Mild	0	28
3	Rt. IC	64	Lt	V	5/5	Intact	Mild	2	25
4	Rt. midbrain	36	Lt	V	5/5	Intact	Mild	2	26
5	Lt. pons	61	Rt	V	4/5	Intact	Mild	1	29
6	Rt. pons	70	Lt	V	5/5	Intact	Mild	0	29
7	Lt. putamen	35	Rt	V	5/5	Intact	Mild	0	29
8	Rt. pons	61	Lt	VI	4/5	Intact	Mild	2	29
9	Lt. putamen	66	Rt	V	5/5	Intact	Mild	2	29
10	Lt. thalamus	27	Rt	VI	5/5	Intact	Mild	0	30

BRS: Brunnstrom stage, MCP: 2nd Metacarpophalangeal joint, CBS: Catherine Bergego Scale, MMSE: Mini-Mental State Examination, IC: internal capsule, Rt: Right, Lt: Left.

Participants’ motor deficits were assessed according to the Brunnstrom stage. A preliminary experiment confirmed that only patients diagnosed with Brunnstrom stage 5 or 6, indicating they were capable of performing horizontal movements in their paretic upper limb, were able to complete the horizontal tracing movement task used in the present study. Therefore, only such patients were included in the main experiment. Proprioceptive and tactile sensations of the upper limb were evaluated by a clinical expert to assess sensory deficits. The proprioceptive sensation assessment passively moved each of two joints (shoulder and 2nd metacarpophalangeal) back and forth in a plane and patients reported segment orientation (i.e., up-down test). The test was repeated six times. Patients who responded without errors were rated “intact” for proprioceptive sensation; patients unable to respond with confidence or with one error were rated “mild.” Patients who were unable to respond or made two or more errors would have been rated “absent” for proprioceptive sensation, but there were no patients whose proprioceptive sensation was rated “absent.” In the tactile sensation assessment, patients responded "yes" when the clinical expert touched their upper limb. This assessment was repeated five times and the total number of correct responses comprised the overall score. A summary of patients’ data is shown in Table 1.

To investigate the impact of sensorimotor deficits on sense of agency, these patients completed tests with both their paretic as well as their non-paralyzed upper limb. The test order was counterbalanced across patients. Because some healthy elderly individuals did not agree to complete the experiment twice, we consequently requested all healthy controls to perform the experiment once using their right upper limb. The experiment was conducted with the approval of the Ethics Committee of the Jinjukai Ishikawa Hospital (2018–1). Each participant provided written informed consent.

### Apparatus

**Fig 2:**
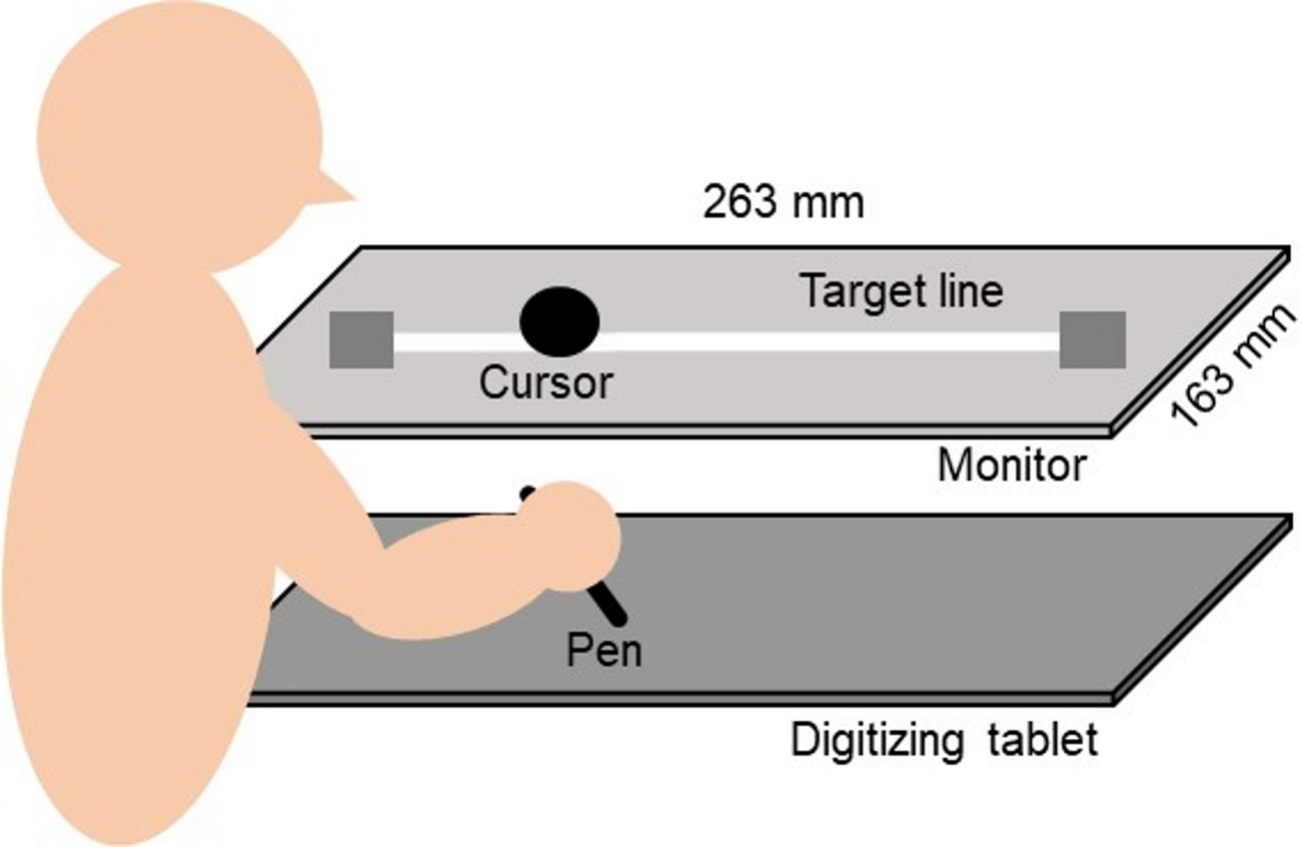
Experimental setup.

A monitor with a 60 Hz refresh rate (CF-SV7TBAQP, Panasonic) was set 20 cm vertically above a digitizing tablet (Intuos Pro Large PTH-851/K, Wacom), as shown in Fig 2. The size of the digitizer input area was set to be approximately equivalent to the plotting area of the monitor (263 × 163 mm). A straight line (length: 230 mm) was horizontally displayed as a target line in the middle of the monitor. The experiment was programmed using Hot Soup Processor 3.4 (Onion Software).

### Procedure

To examine sense of agency in post-stroke patients in terms of motor control, the basic procedure mimicked Asai’s study [16], except that we simplified the procedure so that post-stroke patients could complete the experiment with their paretic upper limb. Participants traced the target line of horizontal movement by manipulating a pen device on the digitizer and received its visual feedback as the cursor on the monitor (Fig 2). Participants first placed the pen at the start position (left side of the target line) during the preparation time of 5s. When a computer started to count up from a sound at zero, participants started moving the pen towards the right end of the target line. After the task began, participants performed four cycles of horizontal movement so that the timing of the pen tip reaching the end of the target line (right or left side) matched the counting from 1s to 8s. When post-stroke patients performed the task with their left upper limb, they started the movement from the right side of the start position and finished at the left side. The vertical distances between the pen position and target line, which constituted movement error, were measured as an index of motor performance. Movement error was used to confirm whether post-stroke patients were capable of completing the movement task as accurately as healthy elderly individuals.

This experiment consisted of 20 trials in a random sequence, with 10 trials in each of the two visual feedback conditions of SELF and OTHER. In the SELF condition, participants received visual feedback of their actual movement as the cursor movement. In the OTHER condition, participants received visual feedback of a cursor representing an other’s movement (recorded in a preliminarily experiment). The others’ movements were randomly selected from a dataset of 30 healthy adults’ movements. While participants performed the four cycles of the horizontal movement, they were required to judge whether the cursor movement reflected their own or an other’s movement by referring to the online spatiotemporal consistency between their actual movement and the cursor’s movement. After each trial, participants verbally reported their self-other judgment on a nine-point scale [34] displayed on the monitor, ranging from 9 (completely self-movement) to 1 (completely the other’s movement). Their responses were analyzed as an indicator of self-other attribution.

The cursor was masked during first and last 0.5s to prevent participants from distinguishing between the self and other’s movements by the timing of the start and end of the movements. Participants were instructed, “You must not deliberately stop the movement and deviate the pen position from the target line until you have completed four cycles of the horizontal movement. While you are tracing the target line, you have to judge whether the cursor movement reflects your own or another’s movement by referring to the spatiotemporal consistency between your actual movement and the cursor’s movement.” Our pilot study confirmed that healthy adults were capable of formulating correct self-other attributions of cursor movement (i.e., no significant misattributions were observed) by performing four cycles of the horizontal movement [35]. Before the main experiment, participants were trained to enable the horizontal movement of their upper limb without compensatory movements of the trunk. Moreover, participants were familiarized with the experimental procedure through training. After an experimenter confirmed that participants understood the procedure following sample trials under the SELF and OTHER conditions, the main experiment started.

### Statistical analyses

#### Homoscedasticity and distribution tests

First, the homoscedasticity and distribution of the data were analyzed by Bartlett’s test of homogeneity of variances and Shapiro-Wilk normality test, respectively. A famer test was conducted only for the between-participants data. Although we conducted a two-way ANOVA to examine self-other judgment and identify an interaction term, the homoscedasticity and distribution tests confirmed non-homoscedastic and non-normal data distribution, so we also conducted non-parametric tests in each condition to compare the non-parametric and parametric test results.

#### Motor performance

An analysis of movement errors was conducted to investigate whether post-stroke patients were capable of completing the movement task as accurately as healthy elderly individuals. The average movement error for each cycle was calculated [16]. Moreover, because differences in movement errors between the SELF and OTHER conditions and across cycles were not of interest, we calculated movement error averages between these conditions and across cycles. We analyzed differences in movement errors among paretic and non-paralyzed upper limbs and healthy elderly scores with a one-way between-participants ANOVA.

#### Self-other judgment within post-stroke patients

To quantify participants’ incorrect self-other judgments (i.e., misattributions), we subtracted the mean score from 9 (the correct score) in the SELF condition and 1 from the mean score of the OTHER condition (i.e., the difference between the correct score and actual score was calculated). Three analyses were conducted to test self-other judgment within post-stroke patients. First, a 2 × 2 within-participants ANOVA analyzed differences in the incorrect responses between the paretic and non-paralyzed upper limbs scores and between the SELF and OTHER conditions.

Second, to examine the relationship between the severity of motor deficits and misattribution, post-stoke patients were divided into two groups based on whether their Brunnstrom stages were 5 or 6 and the Wilcoxon rank sum exact test was used in each of the SELF and OTHER conditions. (We selected a non-parametric test for this analysis because of the small number of participants in each group.)

Finally, to investigate potential lesion site influence, post-stroke patients were divided into two groups depending on whether they had supratentorial or infratentorial lesions. These lesion categories were adopted because there were too few participants for each lesion site and because we wanted to investigate the influences of higher cognitive impairments, which are associated with supratentorial lesions more than infratentorial lesions. If impairments of the higher cognitive functions can affect self-other judgments in this study, the misattributions in patients with a supratentorial lesion would be greater than those in patients with an infratentorial lesion. Although only patients with a subcortical stroke were included in this study, we further investigated this possibility by applying the Wilcoxon rank sum exact test in each of the SELF and OTHER conditions.

#### Self-other judgment between post-stroke patients and healthy elderly

To analyze differences in incorrect responses between post-stroke patients and healthy elderly, we calculated the average of the incorrect responses from the paretic and non-paralyzed upper limb scores and applied a 2 × 2 mixed design ANOVA to analyze the differences.

#### Additional analyses

The experiment was completed twice because post-stroke patients completed the experiment using both their paretic and non-paralyzed upper limbs, whereas healthy elderly individuals completed the experiment using their right upper limb only. To investigate the influence of this difference, two additional analyses were conducted: one analysis compared the results of the first experiment between post-stroke patients and healthy elderly individuals. The other analysis compared the results from the right upper limb of post-stroke patients to those of healthy elderly individuals. Both analyses used a 2 × 2 mixed design ANOVA.

## Results

### Motor performance

**Fig 3:**
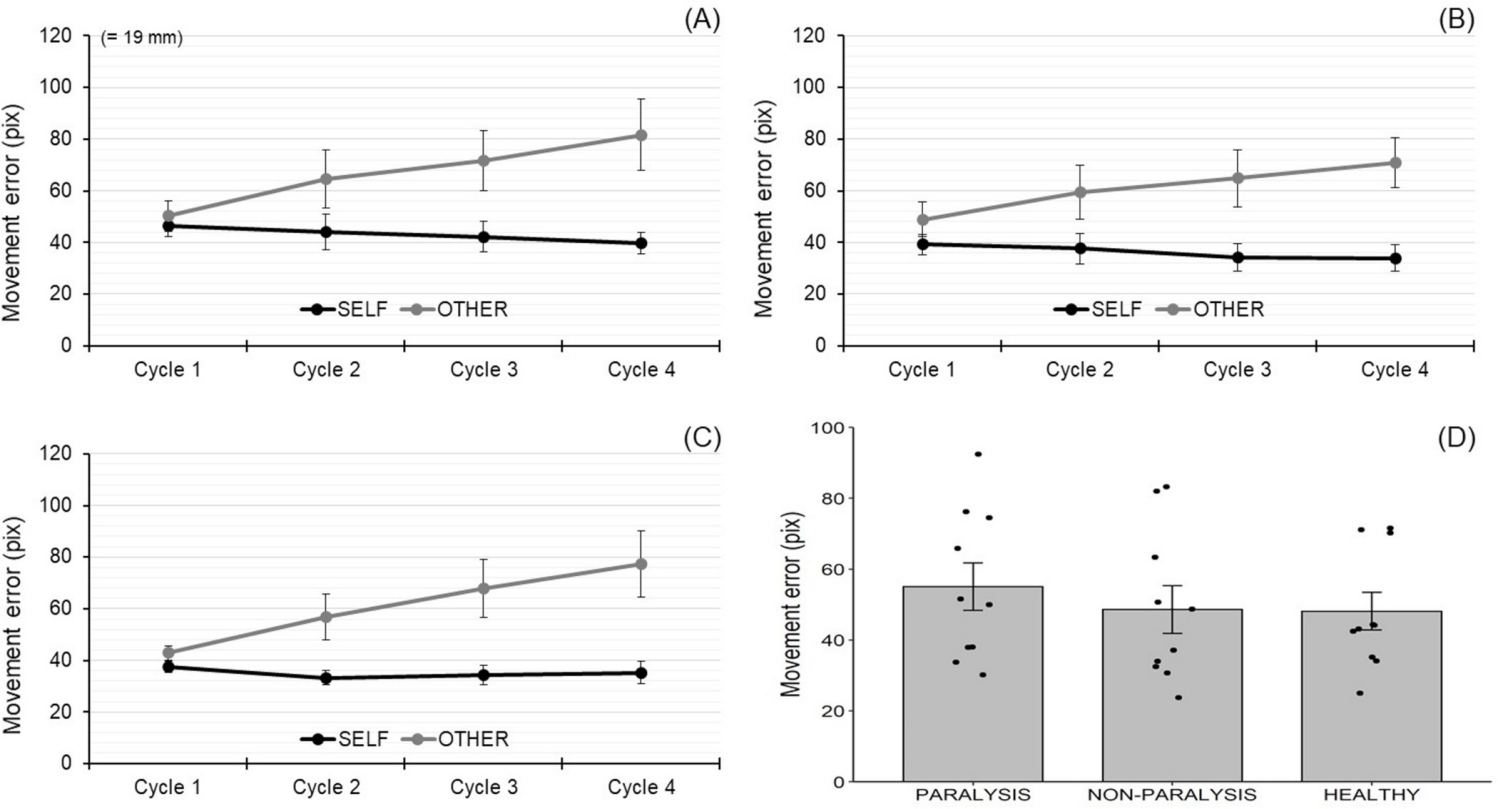
Movement error for paretic upper limb (A), non-paralyzed upper limb (B), healthy elderly individuals (C), and groups (D). For each group, the average of movement errors between the SELF and OTHER conditions and among all cycles was calculated (D). Error bars indicate standard errors.

The Bartlett test (*K*^2^ = .58, df = 2, *p* = .75) and Shapiro-Wilk normality test (*W* = .92, *p* = .38 in the PARALYSIS, *W* = .89, *p* = .16 in the NON-PARALYSIS, *W* = .85, *p* = .06 in the HEALTHY) were not significant. A one-way between-participants ANOVA with a factor of group (PARALYSIS, NON-PARALYSIS, and HEALTHY) revealed no significant differences across groups, *F*(2, 27) = .38, *p* = .69, η_p_^2^ = .027 (Fig 3).

### Self-other judgment within post-stroke patients

**Fig 4:**
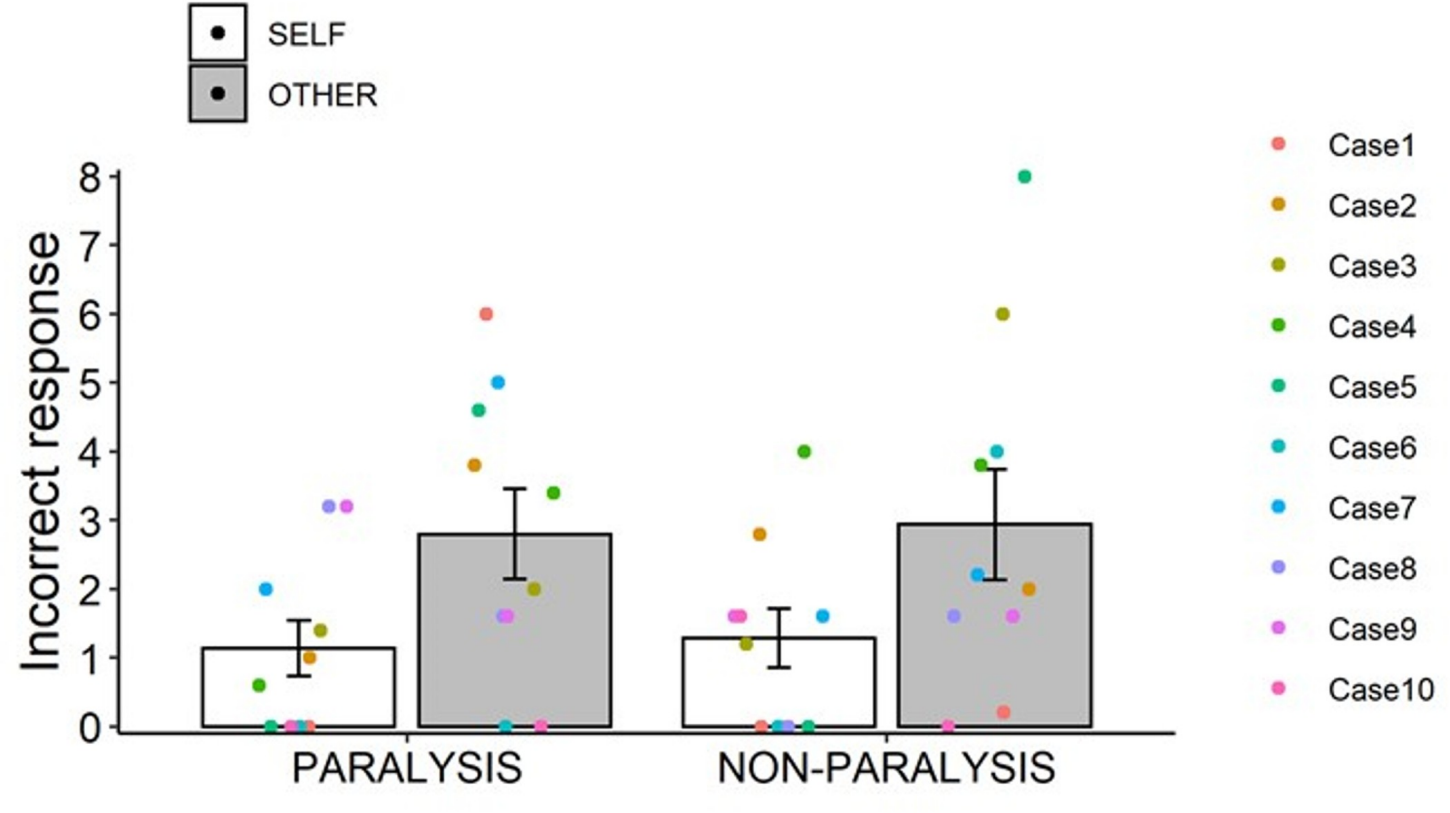
Incorrect responses (i.e., misattributions) on self-other judgments between the paretic and non-paralyzed upper limbs scores in post-stroke patients. In the SELF condition, incorrect responses indicated the other-attribution of self-movement, i.e., participants judged their own movement as an other’s movement. In the OTHER condition, incorrect responses indicated the self-attribution of an other’s movement, i.e., participants judged an other’s movement as their own movement. Error bars indicate standard errors.

Three analyses were conducted to test self-other judgment in post-stroke patients. First, we analyzed differences in the incorrect responses between the paretic and non-paralyzed upper limbs scores (Fig 4). For the paretic upper limbs scores, the Shapiro-Wilk normality test was significant in the SELF condition (*W* = .83, *p* = .03) and not significant in the OTHER condition (*W* = .94, *p* = .59). For the non-paralyzed upper limbs scores, the Shapiro-Wilk normality test was not significant in the SELF (*W* = .86, *p* = .07) or OTHER (*W* = .92, *p* = .33) conditions. Since this test confirmed non-normal data distribution, we conducted a non-parametric test after we considered an interaction term using a two-way ANOVA. A 2 × 2 within-participants ANOVA with factors of upper limb (PARALYSIS and NON-PARALYSIS) and movement (SELF and OTHER) revealed a significant main effect of movement, *F*(1, 9) = 6.16, *p* = .035, η_p_^2^ = .41, indicating that the number of incorrect responses in the OTHER condition was significantly higher than in the SELF condition. The interaction term, *F*(1, 9) = .00, *p* = 1.00, η_p_^2^ = .00, and upper limb main effect, *F*(1, 9) = .06, *p* = .81, η_p_^2^ = .01, were not significant. The Wilcoxon rank sum exact test showed no significant differences between the PARALYSIS and NON-PARALYSIS in the SELF (*W* = 47, *p* = .83) or OTHER (*W* = 48.5, *p* = .93) conditions. These results were consistent with the ANOVA results.

**Fig 5:**
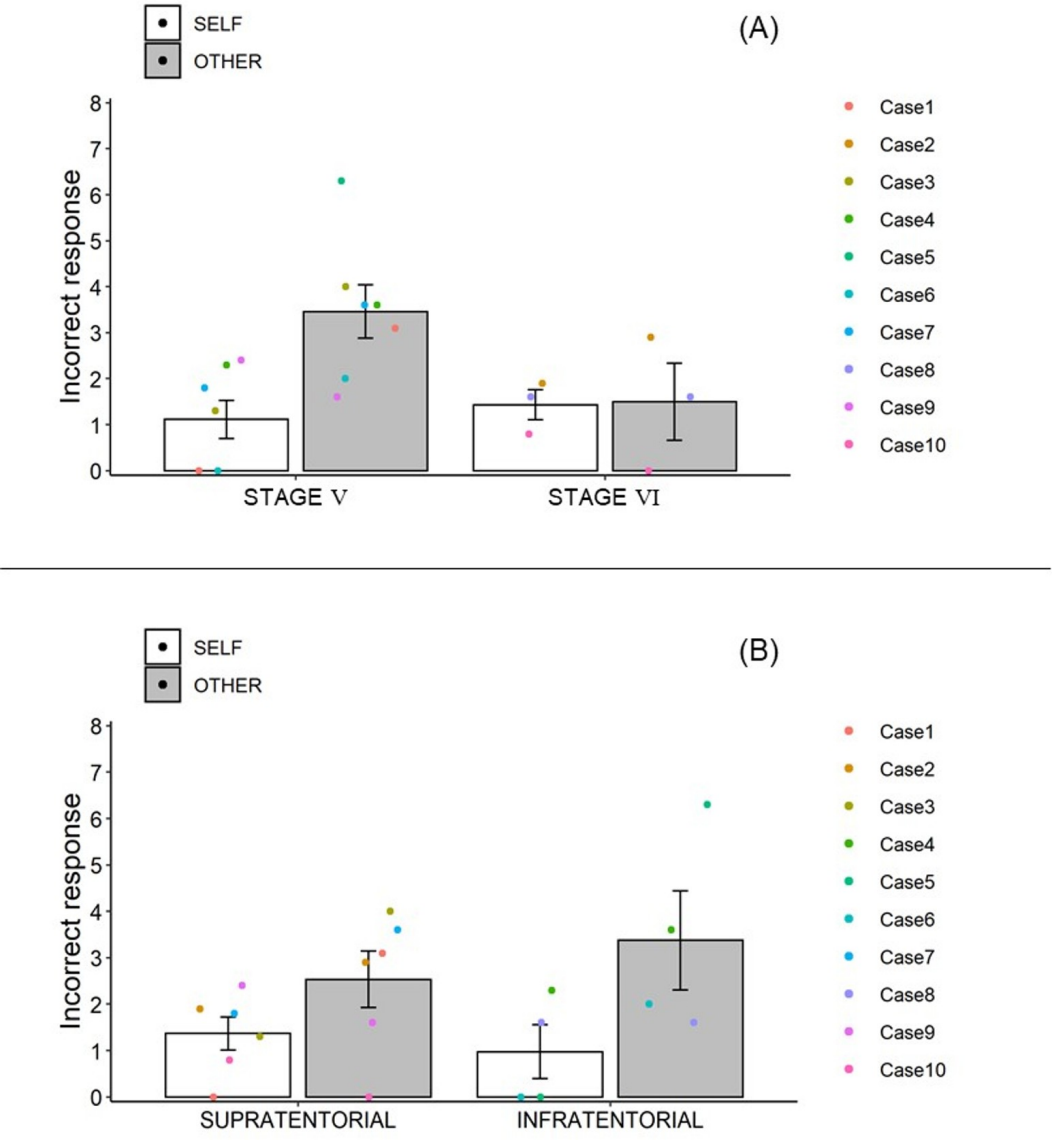
Incorrect responses (i.e., misattributions) on the self-other judgments between post-stroke patients with Brunnstrom stage 5 and 6 (A) and between post-stoke patients with supratentorial and infratentorial lesions (B). Error bars indicate standard errors.

Second, we investigated the relationship between motor deficit severity and misattributions (Fig 5). There were seven patients with Brunnstrom stage 5 and three with Brunnstrom stage 6 (see Table 1). The Wilcoxon rank sum exact test showed no significant differences between the stage 5 and stage 6 groups in the SELF (*W* = 9, *p* = .83) or OTHER (*W* = 18.5, *p* = .075) conditions.

Finally, to investigate the possibility that the impairments in higher cognitive functions affected self-other judgments in post-stoke patients, we compared the incorrect responses in patients with supratentorial lesions to those with infratentorial lesions (Fig 5). Six patients had supratentorial lesions and four had infratentorial lesions (see Table 1). The Wilcoxon rank sum exact test showed no significant differences between the supratentorial and infratentorial groups in the SELF (*W* = 15, *p* = .61) or OTHER (*W* = 10, *p* = .074) conditions, indicating that this result did not support the possibility that lesion type significantly affected self-other judgements.

### Self-other judgment between post-stroke patients and healthy elderly

**Fig 6:**
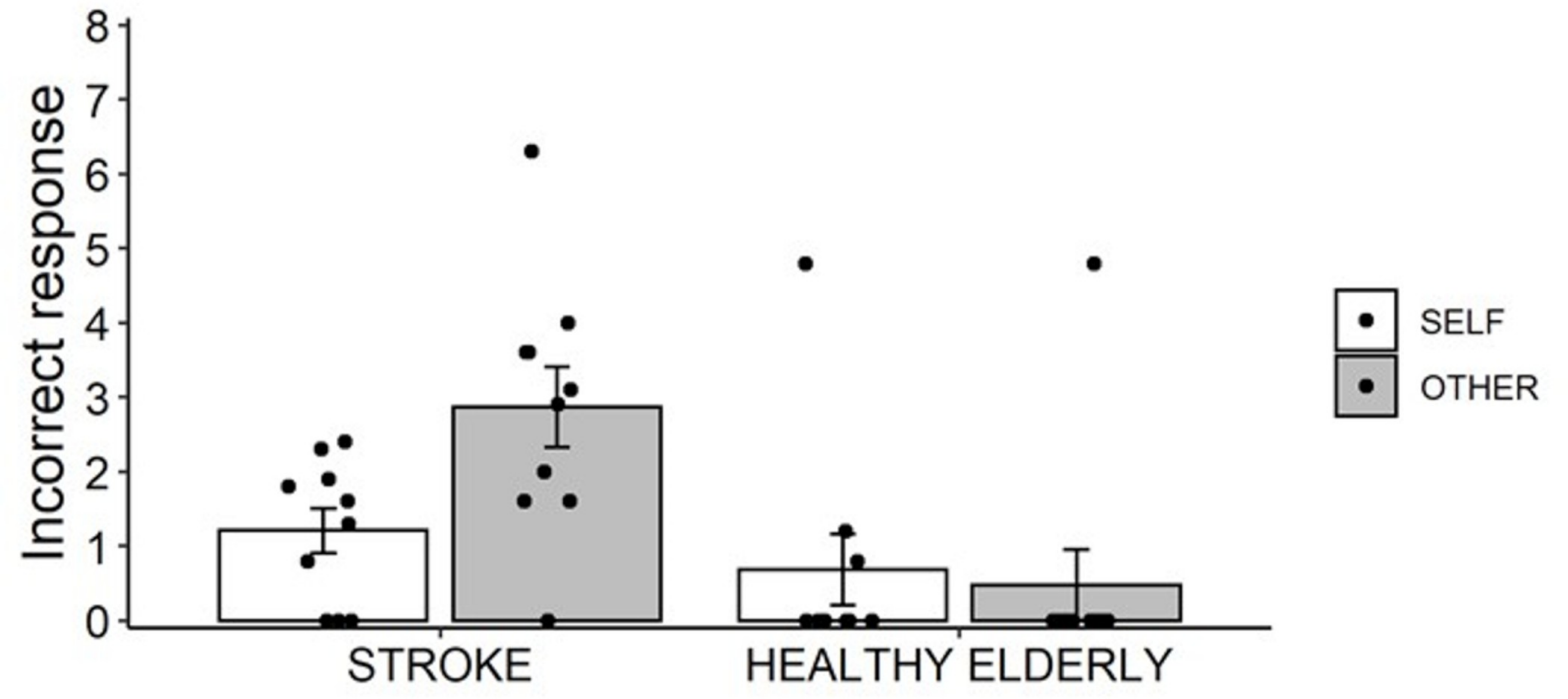
Incorrect responses (i.e., misattributions) in the self-other judgments of post-stroke patients and healthy elderly individuals. In post-stroke patients, differences in the average number of incorrect responses between the paretic and non-paralyzed upper limbs were calculated. The error bars indicate standard errors.

The Bartlett test was not significant (*K*^2^ = 2.95, df = 3, *p* = .40). Regarding the post-stroke patients scores, the Shapiro-Wilk normality test was not significant in the SELF (*W* = .88, *p* = .13) and OTHER (*W* = .96, *p* = .81) conditions. Regarding the healthy elderly scores, the Shapiro-Wilk normality test was significant in the SELF (*W* = .54, *p* < .001) and OTHER (*W* = .37, *p* < .001) conditions. Since this test confirmed the non-normal distribution in the healthy elderly data, we conducted a non-parametric test after we considered an interaction term by a two-way ANOVA. A 2 × 2 mixed design ANOVA with factors of group (STROKE and HEALTHY) and movement (SELF and OTHER) revealed a significant two-way interaction, *F*(1, 18) = 7.43, *p* = .014, η_p_^2^ = .29 (Fig 6). A simple effect analysis revealed that the number of incorrect responses in the OTHER condition was significantly higher than in the SELF condition in the STROKE group, *F*(1, 9) = 6.16, *p* = .035, η_p_^2^ = .41, and the OTHER condition in the HEALTHY group, *F*(1, 18) = 10.91, *p* = .004, η_p_^2^ = .38. Moreover, no significant differences were observed between the STROKE and HEALTHY groups in the SELF condition, *F*(1, 18) = .88, *p* = .36, η_p_^2^ = .05, and between the SELF and OTHER conditions in the HEALTHY group, *F*(1, 9) = 2.14, *p* = .18, η_p_^2^ = .19. Regarding the differences between the STROKE and HEALTHY groups, the Wilcoxon rank sum exact test showed no significant differences in the SELF condition (*W* = 72, *p* = .08) and a significant difference in the OTHER condition (*W* = 86.5, *p* = .002). These results were consistent with those of ANOVA.

Taken together, the results of the self-other judgment analyses show that post-stroke patients, compared with healthy elderly individuals, made significant incorrect self-attributions of others’ movements.

### Additional analyses

**Fig 7:**
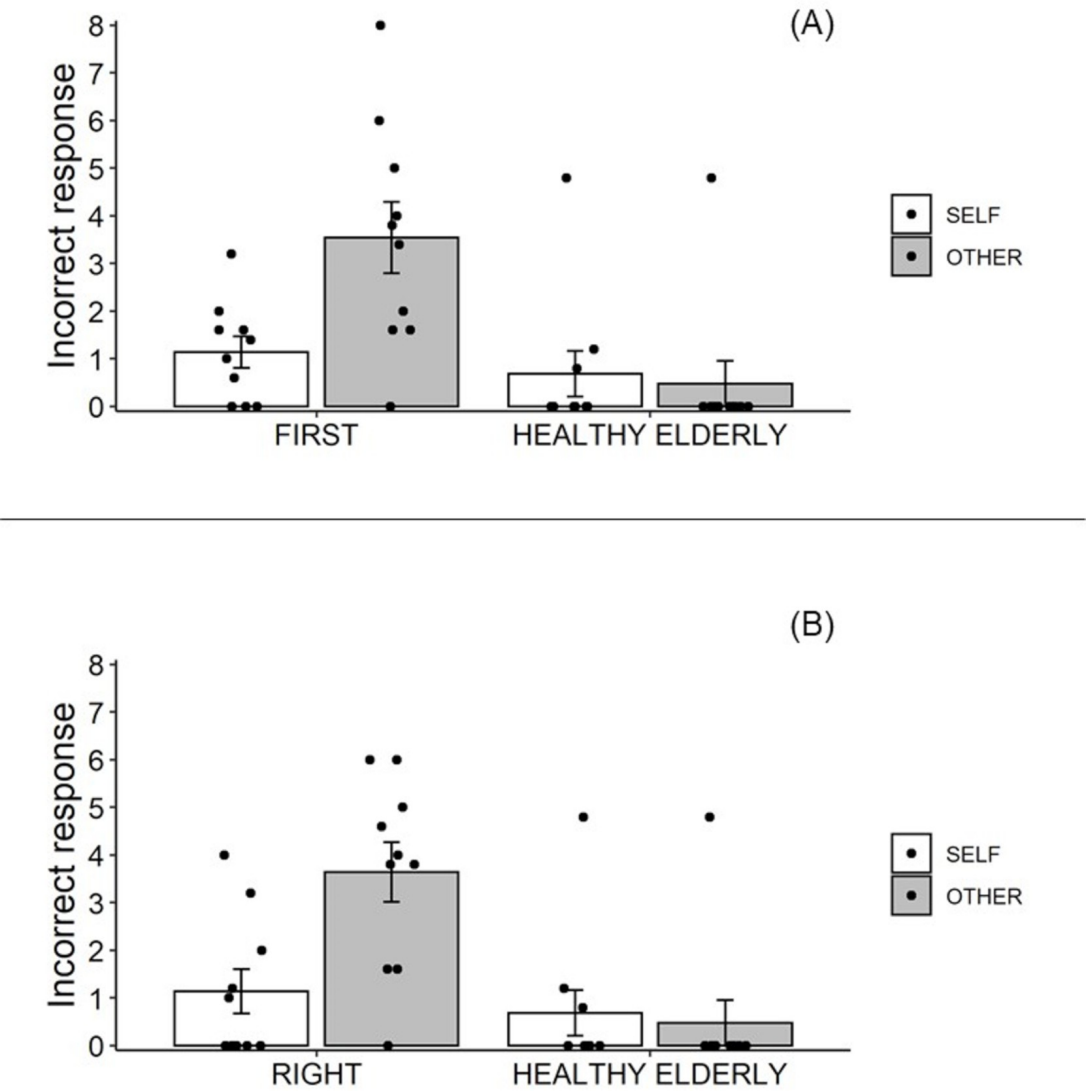
Incorrect responses (i.e., misattributions) in the self-other judgments of post-stroke patients and healthy elderly individuals. In the FIRST (A), the result of the first experiment completed by post-stroke patients was used. In the RIGHT (B), the result of the experiment completed by post-stroke patients with their right upper limb was used. The error bars indicate standard errors.

Two additional analyses were conducted. One compared the first experiment results completed by post-stroke patients to those of healthy elderly individuals (Fig 7). Since a non-normal distribution was detected in healthy elderly data (see “self-other judgment between post-stroke patients and healthy elderly”), we conducted a non-parametric test after we considered an interaction term with a two-way ANOVA. A 2 × 2 mixed design ANOVA with factors of group (FIRST and HEALTHY) and movement (SELF and OTHER) revealed a significant two-way interaction, *F*(1, 18) = 6.74, *p* = .018, η_p_^2^ = .27. A simple effect analysis revealed that the number of incorrect responses in the OTHER condition was significantly higher than in the SELF condition in the FIRST group, *F*(1, 9) = 5.85, *p* = .039, η_p_^2^ = .39, and the OTHER condition in the HEALTHY group, *F*(1, 18) = 11.78, *p* = .003, η_p_^2^ = .40. Moreover, no significant differences were observed between the FIRST and HEALTHY groups in the SELF condition, *F*(1, 18) = .63, *p* = .44, η_p_^2^ = .03, or between the SELF and OTHER conditions in the HEALTHY group, *F*(1, 9) = 2.14, *p* = .18, η_p_^2^ = .19. Regarding the differences between the FIRST and HEALTHY groups, the Wilcoxon rank sum exact test showed no significant differences in the SELF condition (*W* = 70.5, *p* = .10) and a significant difference in the OTHER condition (*W* = 88.5, *p* < .001). These results were consistent with the ANOVA results.

Another analysis compared the results of the experiment completed using the right upper limb of post-stroke patients to those of healthy elderly individuals (Fig 7). A 2 × 2 mixed design ANOVA with factors of group (RIGHT and HEALTHY) and movement (SELF and OTHER) revealed a significant two-way interaction, *F*(1, 18) = 11.43, *p* = .003, η_p_^2^ = .39. A simple effect analysis revealed that the number of incorrect responses in the OTHER condition was significantly higher relative to the SELF condition in the RIGHT group, *F*(1, 9) = 10.10, *p* = .011, η_p_^2^ = .53, and the OTHER condition in the HEALTHY group, *F*(1, 18) = 15.93, *p* < .001, η_p_^2^ = .47. Moreover, no significant differences were observed between the RIGHT and HEALTHY groups in the SELF condition, *F*(1, 18) = .47, *p* = .50, η_p_^2^ = .03, or between the SELF and OTHER conditions in the HEALTHY group, *F*(1, 9) = 2.14, *p* = .18, η_p_^2^ = .19. Regarding the differences between the RIGHT and HEALTHY groups, the Wilcoxon rank sum exact test showed no significant differences in the SELF condition (*W* = 61, *p* = .38) and a significant difference in the OTHER condition (*W* = 88.5, *p* < .001). These results were consistent with those of the ANOVA.

The results of two additional analyses were similar to that of the main analysis between the STROKE and HEALTHY groups, suggesting that the significant misattribution of the other’s movement in post-stroke patients was not associated with the number of the completed experiments or which upper limb was used.

## Discussion

Using a self-other attribution task, the present study explored the relationship between sense of agency and sensorimotor deficits by investigating agency judgments in post-stroke patients. These patients had damage located in subcortical sensorimotor-related regions such as the internal capsule or pons, aside from cortical areas. Regarding the results of motor performance, there were no significant differences among scores relating to paretic upper limb usage, non-paralyzed upper limb usage, and those of healthy elderly individuals. This result suggests that the level of difficulty that resulted from simplifying the task was suitable for study participants. Moreover, this result does not support the possibility that post-stroke patients failed to correctly perform the task. In the self-other judgments, the results showed that in comparison to healthy elderly individuals, post-stroke patients made significant incorrect self-attributions of others’ movements. Interestingly, these significant misattributions were observed even when participants used the non-paralyzed upper limb. We hypothesized that if the post-stroke patients had impaired prediction error detection resulting from their sensorimotor deficits, the misattributions should only be observed in performances involving their paretic upper limbs. However, there were no significant differences between paretic and non-paralyzed upper limb performance, which did not support our hypothesis. Some studies have suggested that the parietal region plays an important role in sensorimotor information comparisons, such as motor intention, internal prediction, and sensory feedback [36–39]. For example, in an experimental study of patients with parietal lesion induced apraxia, participants made misattributions irrespective of whether their upper limbs were paralyzed [29]. In the present study, however, only the patients with no lesions in the parietal, insula, and prefrontal cortices were recruited. Therefore, features other than error detection need to be examined.

Synofzik [40] posited that there are two aspects to agency registration. One aspect refers to the sense of agency produced through sensorimotor information, including internal prediction and sensory feedback. Previous studies have suggested that sense of agency is predominantly based on error detection in the comparison between internal prediction and sensory feedback [14,16,41]. A second aspect refers to the sense of agency that emerges in the case when the available cognitive information (e.g., ideas, knowledge, or beliefs) is consistent with the outcome of the action [42–45]. If a person has a thought consistent with a subsequent action, that person can experience an illusory sense of agency despite that the person is not the author of that action [46–48]. In the present study, since participants were required to trace a horizontal line, they should have believed that they performed that action. Moreover, the cursor moved horizontally in the same manner that it had for the participants. In such a situation, participants’ thoughts concerning the horizontal movement should have been consistent with the cursor’s horizontal movement, despite that the spatiotemporal motor properties between the participants’ actual tracings and cursor movements did not match. Indeed, the significant misattributions (i.e., illusory self-attribution) were observed only in the OTHER condition (self-attribution of the cursor was the correct response in the SELF condition). If the post-stroke patients had impaired error detection related to a corrupted sensorimotor system, given the difficulty in comparing actual and cursor movements, they could be expected to make significant misattributions in both the SELF and OTHER conditions. The cognitive aspect of agency may explain why patients made significant misattributions in the performance of each upper limb in the OTHER condition only.

However, it remains unclear why post-stroke patients with sensorimotor deficits made self-other attributions based on cognitive, as opposed to sensorimotor, information. Given that the present study did not manipulate any cognitive factors, the aforementioned explanation still remains only one of many possibilities. To examine this possibility, a continuous study that manipulates cognitive information in self-other attributions should be conducted. For this continuous study, we formulated a suggestion based on cue integration theory, which proposes that the weight of an agency cue in self-other attribution varies according to its relative reliability in a given situation [49–52]. Usually, internal prediction is the most reliable information in respect to the registration of agency [51]. However, when sensorimotor information is considerably noisy, it is less reliable because it is more difficult to compare internal predictions with the sensory feedback. Accordingly, other agency cues may be given more weight [53].

Interestingly, similar findings have been demonstrated in studies of schizophrenic patients, who typically have difficulty distinguishing between their own actions and the actions of others [54–58]. Previous studies have suggested that schizophrenia is characterized by impaired internal predictions, which result in misattributions [59–61]. Importantly, several studies revealed that self-other attributions in schizophrenic patients are based on external agency cues instead of internal predictions [60,61]. Cue integration theory provides an explanation for these results, which suggests that owing to impaired prediction, external information is given more weight than internal signals [49,51,62]. Given their sensorimotor deficits, post-stroke patients receive less or noisier sensorimotor information in their daily lives. According to cue integration theory, under such conditions, self-other attribution in post-stroke patients may well be based on a different attribution strategy than that used by healthy people. In other words, post-stroke patients with sensorimotor deficits may begin to depend on cognitive information in place of sensorimotor information.

Several limitations in the present study require consideration. To examine the influences of sensorimotor deficits, post-stroke patients were required to complete the experiment using both their paretic and non-paralyzed upper limbs. Therefore, we were unable to recruit patients with severe paralysis (i.e., Brunnstrom stage 4 or under). Regarding the misattributions between post-stroke patients with Brunnstrom stage 5 and 6, significant differences were not observed. This may be because of the small sample size (there were just three patients with Brunnstrom stage 6). Moreover, it was necessary to reduce the number of trials in the experiment. Given that there were no significant differences in the results between the upper limbs, in future research it may be worth focusing solely on the performance of non-paralyzed upper limbs in this task paradigm. This would permit conducting an experiment involving a sufficient number of trials and allowing inclusion of patients with severe paralysis. Furthermore, recruiting patients with severe paralysis would contribute to producing a greater sample size, allowing investigators to examine the relationship between misattribution and sensorimotor deficit severity.

In summary, the present study showed that post-stroke patients with sensorimotor deficits made incorrect self-attributions of others’ movements in agency judgments. This incorrect agency judgment was observed in both upper limb types (paretic and non-paralyzed). Post-stroke patients might have made self-other attributions based on the consistency between their thoughts and the cursor movement rather than the spatiotemporal consistency between their actual movement and the cursor movement. Cue integration theory asserts that self-other attribution for post-stroke patients with sensorimotor deficits might depend on cognitive information, such as thoughts or knowledge, rather than sensorimotor information, including internal prediction, because of their sensorimotor deficits. A further study investigating whether and why post-stroke patients are potentially dependent on cognitive information in agency registration should be conducted.

## Supporting information

S1 File Supporting information file. (XLSX)
